# ADAM9 enhances CDCP1 protein expression by suppressing miR-218 for lung tumor metastasis

**DOI:** 10.1038/srep16426

**Published:** 2015-11-10

**Authors:** Kuo-Liang Chiu, Ting-Ting Kuo, Qian-Yu Kuok, Yu-Sen Lin, Chung-Hung Hua, Chen-Yuan Lin, Pei-Yuan Su, Liang-Chuan Lai, Yuh-Pyng Sher

**Affiliations:** 1 Graduate Institute of Clinical Medical Science, China Medical University, Taichung 404, Taiwan; 2 Division of Chest Medicine, Department of Internal Medicine, Taichung Tzu-Chi Hospital, Buddhist Tzu Chi Medical Foundation, Taichung 427, Taiwan; 3 School of Post-baccalaureate Chinese Medicine, Tzu Chi University, Hualien 970, Taiwan; 4 Center for Molecular Medicine, Tzu Chi University, Hualien 970, Taiwan; 5 Division of Thoracic Surgery, Tzu Chi University, Hualien 970, Taiwan; 6 Department of Otolaryngology, Tzu Chi University, Hualien 970, Taiwan; 7 Division of Hematology and Oncology, China Medical University Hospital, Taichung 404, Taiwan; 8 Gastroenterology and Hepatology, Department of Internal Medicine, Changhua Christian Hospital, Changhua, Taiwan; 9 Graduate Institute of Physiology, National Taiwan University, Taipei 106, Taiwan

## Abstract

Metastasis is the leading cause of death in cancer patients due to the difficulty of controlling this complex process. MicroRNAs (miRNA), endogenous noncoding short RNAs with important biological and pathological functions, may play a regulatory role during cancer metastasis, but this role has yet to be fully defined. We previously demonstrated that ADAM9 enhanced the expression of the pro-migratory protein CDCP1 to promote lung metastasis; however, the regulatory process remains unknown. Here we demonstrate that endogenous miR-218, which is abundant in normal lung tissue but suppressed in lung tumors, is regulated during the process of ADAM9-mediated CDCP1 expression. Suppression of miR-218 was associated with high migration ability in lung cancer cells. Direct interaction between miR-218 and the 3′-UTR of *CDCP1* mRNAs was detected in luciferase-based transcription reporter assays. CDCP1 protein levels decreased as expression levels of miR-218 increased, and increased in cells treated with miR-218 antagomirs. Induction of miR-218 inhibited tumor cell mobility, anchorage-free survival, and tumor-initiating cell formation *in vitro* and delayed tumor metastases in mice. Our findings revealed an integrative tumor suppressor function of miR-218 in lung carcinogenesis and metastasis.

Lung cancer is the leading cause of death from cancer worldwide, due to high metastatic rates even when patients are diagnosed at early stages^1^. The 5-year overall survival rate of lung cancer is only 15% despite several advanced therapeutic strategies that have been developed during recent decades^2^. Lung cancer patients exhibit a high incidence of brain metastasis, approximately 40–50%, during the course of their disease^3^. Most lung cancer patients with brain metastases have multiple lesions, and this is associated with a survival time of 3–6 months. Therefore, to prevent lung cancer metastasis is an important medical issue for prolonging the survival time of lung cancer patients.

Several genes are reported to be associated with lung cancer metastasis, such as *ADAM9* (a disintegrin and metalloprotease 9) and *CDCP1* (CUB-domain-containing protein 1). ADAM9, a type I transmembrane protein of the ADAM family, is involved in cell adhesion and migration via its disintegrin domain for adhesion and its metalloproteinase domain for ectodomain shedding^4^,^5^,^6^. Ectopic expression of ADAM9 in lung cancer cells is correlated with brain metastasis^7^. CDCP1, a cell surface glycoprotein for cell-cell interactions, is overexpressed in many cancers and promotes cancer metastasis to other parts of the body, such as colon cancer and melanoma^8^,^9^. It has been reported as a novel regulator for increasing anoikis resistance in lung adenocarcinomas^10^. Knockdown of CDCP1 blocks tumor metastasis and peritoneal dissemination *in vivo*, without significantly affecting cell proliferation^10^, suggesting CDCP1 is a potential target to disrupt the progression of cancer.

MicroRNAs (miRNA), generally 18–25 nt long, can regulate gene expression^11^,^12^. Misregulation of several miRNAs has been linked to the development of certain human diseases, including cancer. Thus ‘miRNA replacement therapy’, which involves introducing miRNAs into diseased tissues, is viewed as a potential therapeutic approach^13^,^14^.

Our previous study demonstrated that ADAM9 promotes lung cancer metastasis by enhancing the function of CDCP1^15^. However, the regulatory mechanisms that activate CDCP1 expression are still unknown. We have reported that *ADAM9* activates *CDH2* (Cadherin 2, N-cadherin) through the release of miR-218 inhibition on *CDH2* in lung adenocarcinoma^16^. Decreased miR-218 expression is frequently observed in various cancers and acts as tumor suppressor by targeting many oncogenes^17^. Ectopic expression of miR-218 reduced cell proliferation, invasion, and migration of lung cancer cells^18^. In this study, we showed that ADAM9 enhances CDCP1 expression by suppressing miR-218 expression. We also demonstrated the therapeutic effect of blocking lung cancer metastases in animal models by restoring dysregulated miRNAs involved in the ADAM9-CDCP1 axis.

## Results

### ADAM9 activates CDCP1 protein expression and enhances cell migration

**Figure 1:**
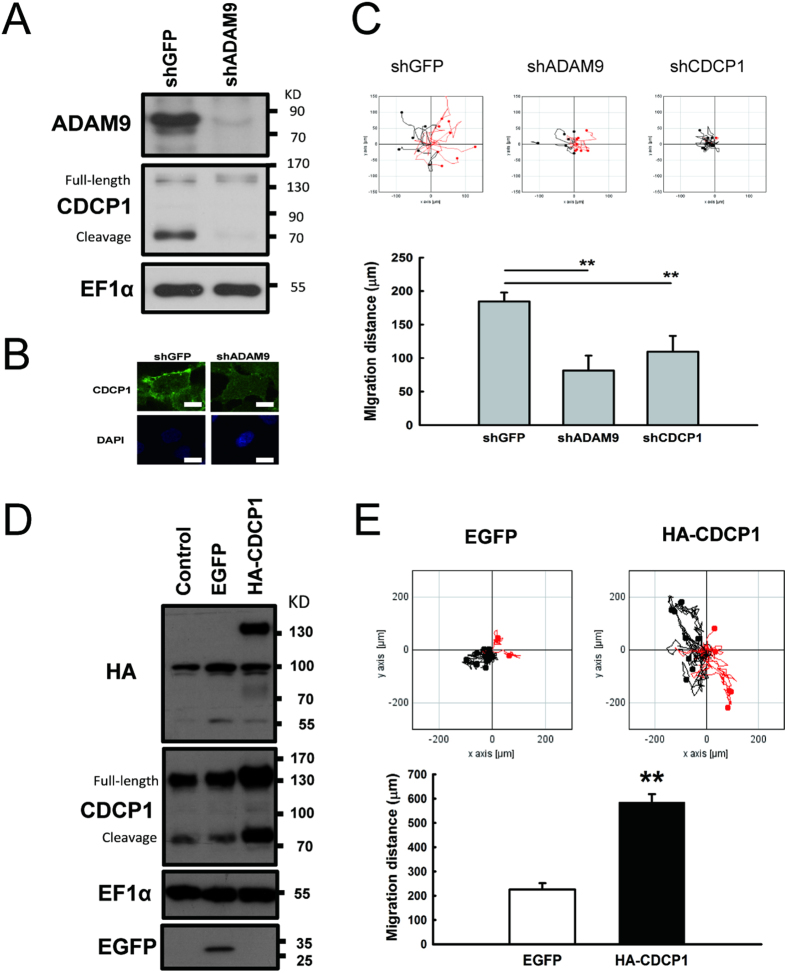
ADAM9 activates CDCP1 protein expression and enhances cell migration. (**A**) Western blot of ADAM9 and CDCP1 in Bm7-shGFP (control) and -shADAM9 cells. ADAM9 proteins were detected in non-reducing gels using the antibody from R&D (MAB939, R&D Systems). CDCP1 proteins contain full-length and proteolytically processed forms. EF1α was used as protein loading control. (**B**) Immunohistochemical analysis of CDCP1 in ADAM9-depleted Bm7 cells. Scale bar: 20 μm. (**C**) Migration ability of Bm7-shGFP, -shADAM9, and -shCDCP1 cells using time-lapse video microscopy. Top: representative graph of motile activity of 15 randomly chosen cells. Migration traces to the right are shown in red, and to the left in black. Bottom: quantitation of migration distance over 16 h from three independent experiments. (**D**) Western blots of F4 cells overexpressing EGFP (F4-EGFP) and HA-CDCP1 (F4-HA-CDCP1) by retrovirus infection. (**E**) Migration ability of individual F4 cell overexpressing EGFP or CDCP1 using time-lapse video microscopy (top). The accumulated migration distance for 16 hours was quantified (bottom). Error bars, SD; **P < 0.01.

Previously, we found that ADAM9-depleted lung cancer cells decreased *CDCP1* expression and cell migration^15^. In order to investigate whether CDCP1 was involved in ADAM9-mediated cell migration, we first examined the relationship between ADAM9 and CDCP1. As shown in Fig. 1, when ADAM9 was knocked down, CDCP1 protein levels including full-length and cleavage forms were decreased (Fig. 1A), and immunofluorescence analysis showed that fewer CDCP1 were observed in membrane (Fig. 1B). Also, silencing ADAM9 or CDCP1 in lung cancer Bm7 cells decreased their migration (Fig. 1C). Next, human influenza hemagglutinin (HA) tagged CDCP1 (HA-CDCP1) was overexpressed in lung cancer F4 cells (Fig. 1D). The cumulative migration distance in CDCP1-overexpressing F4 cells (HA-CDCP1) was significantly farther than that in control F4 cells expressing enhanced green fluorescent protein (EGFP) (Fig. 1E). These results suggested that ADAM9 can enhance lung cancer migration via up-regulating CDCP1.

### Characterization of ADAM9-regulated miRNA that targets CDCP1

**Figure 2:**
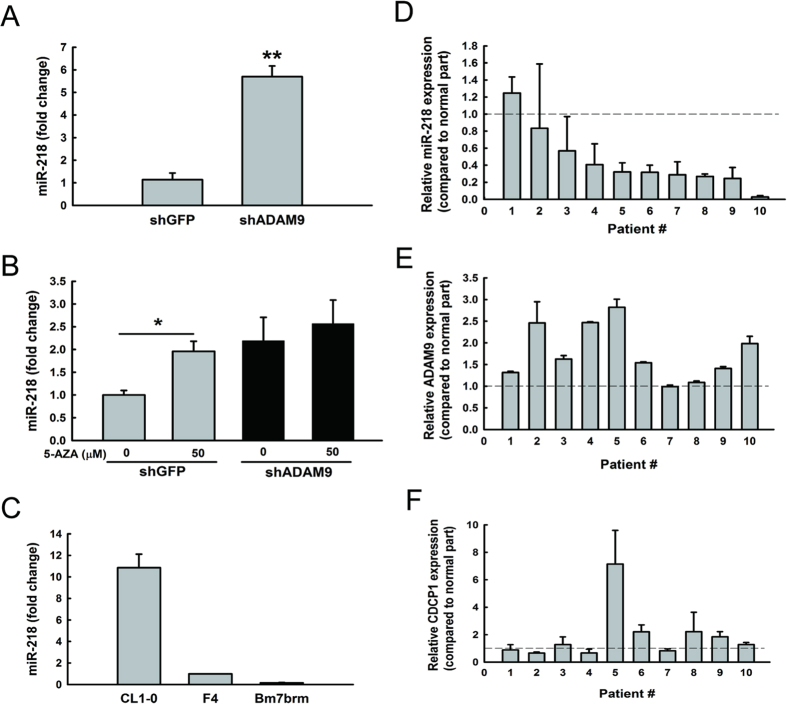
The expression of miR-218 is reduced in lung cancer cells. (**A**) qRT-PCR analysis of miR-218 levels in stable clone of *ADAM9* knockdown cells. (**B**) qRT-PCR analysis of miR-218 expression in Bm7 cells 3 days after treatment with 5-aza. The pooled population by lentivirus expressing shRNAs targeting *ADAM9* was used in this assay. (**C**) qRT-PCR analysis of miR-218 in lung cancer cell lines with progressively greater metastatic ability. (**D**) Relative expression of miR-218, (**E**) *ADAM9*, and (**F**) *CDCP1* in lung cancer tissues from 10 patients, as compared to normal tissue counterparts. *P < 0.05. **P < 0.01.

Next, to investigate which molecule is involved in the process of ADAM9 regulating CDCP1 expression, we hypothesized that miRNAs are involved in this process. Therefore, we examined differentially expressed miRNAs in ADAM9 knockdown cells using Illumina miRNA beadchips and searched miRNAs that were predicted to target *CDCP1* using miRSystem^19^. Of the miRNA candidates, miR-218 was selected after confirmation that its expression levels were significantly (*P* < 0.01) increased in ADAM9 knockdown cells using quantitative miR-RT-PCR (Fig. 2A). Furthermore, using 5-Aza-2′-deoxycytidine (5-AZA) to inhibit DNA methylation, we found that miR-218 expression was significantly (*P* < 0.05) increased in shGFP cells but not in ADAM9 knockdown cells (Fig. 2B), suggesting ADAM9 may inhibit miR-218 expression via DNA methylation.

To examine whether invasion ability was correlated with the levels of miR-218, we examined the endogenous levels of miR-218 in three lung cancer cells with progressive invasion ability (Bm7brm >F4 >CL1-0). The levels of miR-218 were decreased as the invasion ability of lung cancer cell lines increased (Fig. 2C). Lastly, we also examined the endogenous expression levels of miR-218 in 10 primary clinical lung tumor specimens. The majority (80%) of tumor samples had lower expression levels of miR-218 as compared to their normal counterparts; seven tumor samples had less than half the miR-218 expression of their normal tissue counterparts (Fig. 2D). Furthermore, relative expression levels of *ADAM9* and *CDCP1* in tumor compared to normal part were measured among these samples (Fig. 2E,F). *ADAM9* and *CDCP1* are positively correlated (R = 0.44), whereas miR-218 negatively correlated with *ADAM9* (R = −0.01) and *CDCP1* (R = −0.27). Taken together, these results show that miR-218 expression is decreased in lung cancer cells and could be re-induced in ADAM9 knockdown lung cancer cells.

### miR-218 directly regulates CDCP1

**Figure 3:**
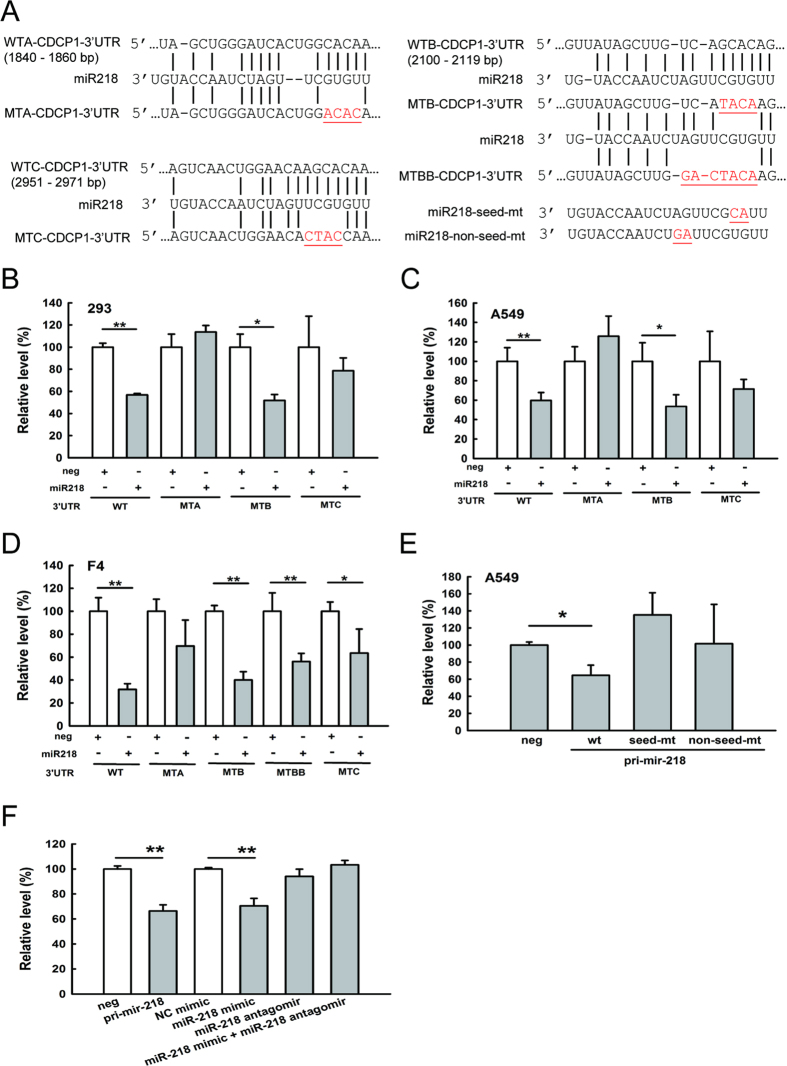
*CDCP1* is a target of miR-218. (**A**) Schematic representation of miR-218 targeting the *CDCP1* 3′-UTR. Three potential miR-218 binding sites are located at 1,840–1,860 bp (site WTA), 2,100–2,119 bp (site WTB), and 2,951–2,971 bp (site WTC) from the transcription start site of *CDCP1*. Mutation sequences at these 3 miR-218 binding sites and miR-218 mutation at seed or non-seed sites are marked with red letters and underlines. (**B–D**) Luciferase assays of miR-218 binding to the wild-type and mutated *CDCP1* 3′-UTR in HEK 293 cells (**B**), A549 cells (**C**), and F4 cells (**D**). Cells were co-transfected with the plasmid of pri-mir-218 as miR-218, the firefly luciferase construct of *CDCP1* 3′-UTR, and the *Renilla* luciferase control for the dual-luciferase assay. The relative luciferase activity represents the dual luciferase activity ratio (firefly/*Renilla* luciferase). WT: wild-type; MTA, MTB, MTC: mutation at site A, B, or C, respectively; MTBB: extended length of mutant sequence at site B. (**E**) Luciferase assays of A549 cells co-transfected with pri-mir-218 wild-type or mutants, the firefly luciferase construct of *CDCP1* 3′-UTR, and the *Renilla* luciferase control. (**F**) Luciferase assays of miR-218 binding to the wild-type *CDCP1* 3′-UTR in A549 cells. Pri-mir-218, miR-218 mimic, or miR-218 antagomir were included in the assays. *P < 0.05. **P < 0.01.

To explore whether miR-218 can bind and regulate *CDCP1*, we used miRSystem to predict binding sites of miR-218 in the *CDCP1* 3′ untranslated region (3′-UTR) and examined their interaction using luciferase assays. Three potential binding sites were predicted at 1,840–1,860 bp, 2,100–2,119 bp, and 2,951–2,971 bp from the transcription start site of *CDCP1*. We mutated these binding sites to determine which binding sites were targeted by miR-218 (Fig. 3A).

**Figure 4:**
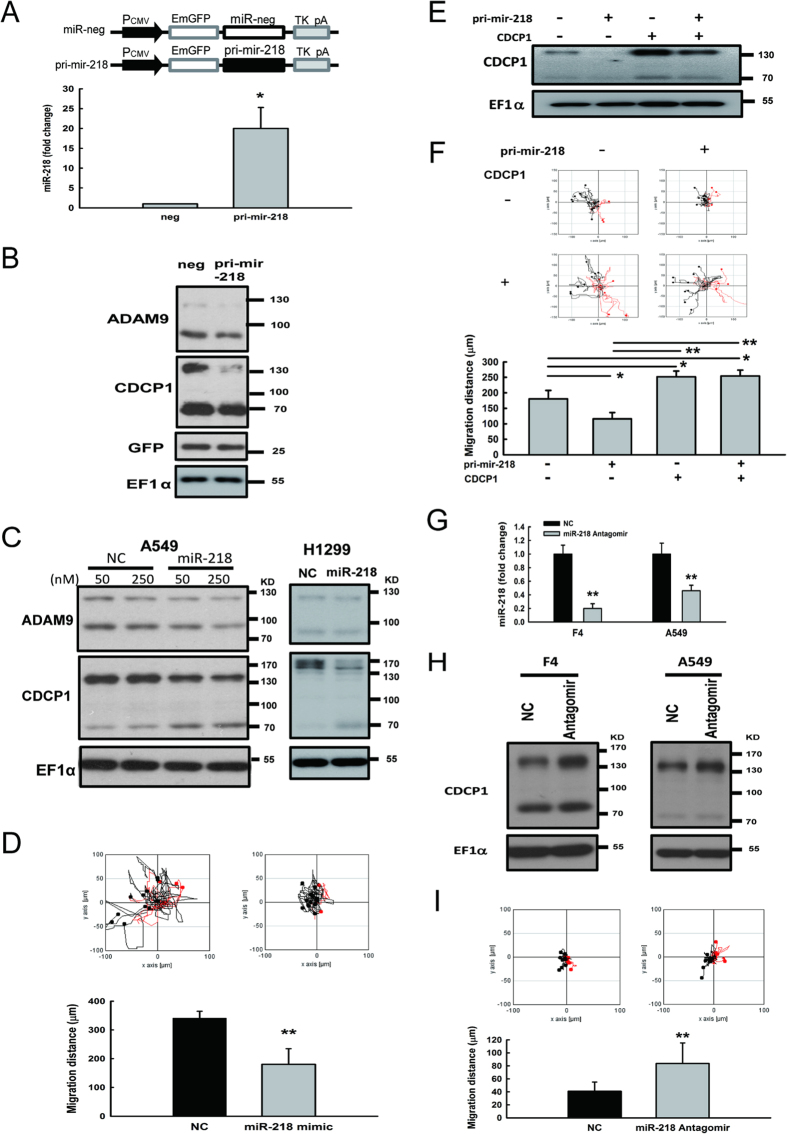
CDCP1 expression is modulated by miR-218. (**A**) qRT-PCR analysis of Bm7 cells overexpressing pri-mir-218. A schematic representation of the pri-mir-218 construct is shown at the top. The expression levels of miR-218 were detected 48 h after transfection. miR-191 was used as an internal control. (**B**) Western blot analysis of CDCP1 in Bm7 cells overexpressing pri-mir-218. EF1α was used as an internal control. **(C**) Western blot analysis of CDCP1 in lung cancer cells treated with miR-218 mimic oligonucleotides. A549 and H1299 cells were administered 50 nM of miR-218 mimic. (**D**) Migration ability of Bm7 cells transfected with miR-218 mimic (50 nM) using time-lapse video microscopy. (**E**) Western blot analysis of CDCP1 in F4 cells overexpressing pri-mir-218 or/and plasmids of *CDCP1* lacking 3′UTR. (**F**) Migration ability of F4 cells transfected with pri-mir-218 or/and plasmids of *CDCP1* lacking 3′UTR using time-lapse video microscopy (top). The 16-hour migration distance was quantified (botton). (**G**) Relative expression levels of miR-218 in lung cancer F4 and A549 cells transfected with miR-218 antagomirs (200 nM). U6B was used as an internal control. NC: negative control. (**H**) Protein levels of CDCP1 in lung cancer F4 and A549 cells transfected with miR-218 antagomirs (200 nM). (**I**) Migration ability of F4 cells transfected with miR-218 antagomirs (200 nM) using time-lapse video microscopy. *P < 0.05. **P < 0.01.

By co-transfecting the miR-218 plasmids (construct shown in Fig. 4A) and the reporter construct, which contained the *CDCP1* 3′-UTR behind the luciferase gene, we showed that miR-218 was better able to inhibit the luciferase activity compared with the empty vector control in HEK 293 cells (Fig. 3B), A549 cells (Fig. 3C), and F4 cells (Fig. 3D). In general, when we mutated binding sites A or C, the luciferase activity was recovered, but it was not recovered when site B was mutated (Fig. 3B–D). To further investigate mutations at site B, we mutated additional nucleotides at site B (MTBB) but still could not relieve the suppression of luciferase activity (Fig. 3D), suggesting site B was not a binding site for miR-218.

In addition, mutations of miR-218 at the seed site or non-seed site were constructed to evaluate which region of miR-218 was essential for targeting *CDCP1* (Fig. 3A). The results showed that, regardless of the mutated sites, both mutations of miR-218 could relieve the suppression of luciferase activity (Fig. 3E). Taken together, these results showed that miR-218 can bind to the 3′-UTR of *CDCP1* at two sites. To further demonstrate that *CDCP1* is the target of miR-218, miR-218 mimic and antagomir were applied in luciferase assays. Similar suppression of luciferase activity was found in cells transduced with pri-mir-218 or treated with miR-218 mimic (Fig. 3F). As expected, luciferase activity did not decrease in cells treated with miR-218 antagomir alone or combination of miR-218 mimic and antagomir (Fig. 3F), indicating the fact that miR-218 targets *CDCP1*.

### CDCP1 expression is regulated by miR-218

To further confirm that *CDCP1* expression could be inhibited by miR-218, we inserted the primary mir-218 sequence behind the EGFP gene sequence and proved that miR-218 can be successfully expressed (Fig. 4A). The protein levels of CDCP1 were decreased in miR-218 overexpressing cells (Fig. 4B). A similar observation was found in lung cancer cells transfected with miR-218 mimic oligomers (Fig. 4C). The migration ability of lung cancer Bm7 cells transfected with miR-218 mimic was significantly inhibited (Fig. 4D). To further demonstrate that the suppressed oncogenic properties of miR-218 directly through target *CDCP1*, western blot analysis and cell migration assays were performed in lung cancer F4 cells over-expressing pri-mir-218 and/or *CDCP1* lacking 3′UTR (Fig. 4E,F). Ectopic expression of *CDCP1* lacking the 3′UTR increased CDCP1 protein expression and remained high level of CDCP1 with co-expression of pri-mir-218 (Fig. 4E). Consistent with the CDCP1 level, ectopic expression of CDCP1 lacking the 3′UTR increased cell migration and over-expression of miR-218 cannot inhibit the mobility of lung adenocarcinoma cells (Fig. 4F).

Conversely, when lung cancer cells were treated with miR-218 antagomirs, levels of miR-218 were decreased (Fig. 4G), but the amount of CDCP1 protein was increased in lung cancer cells at 48 h after miR-218 antagomirs transfection (Fig. 4H). In addition, the migration ability of lung cancer F4 cells transfected with miR-218 antagomirs was significantly increased (Fig. 4I).

### Induction of miR-218 inhibits tumor cell mobility, anchorage-free survival, and tumor sphere formation

**Figure 5:**
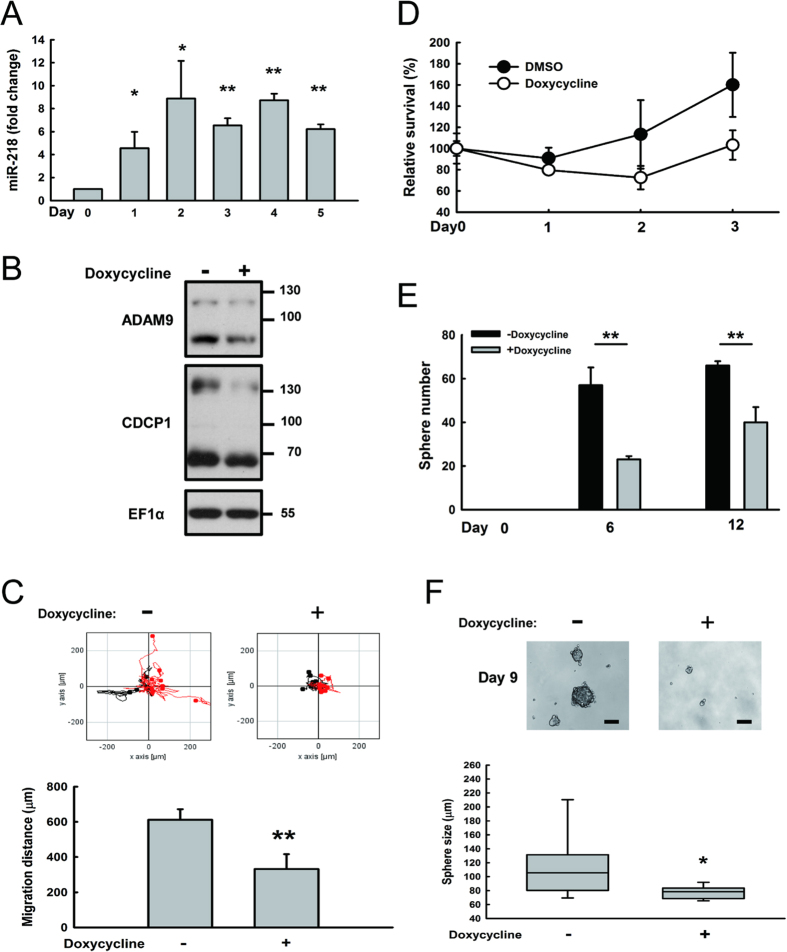
Induction of miR-218 inhibits tumor cell mobility, anchorage-free survival and tumor sphere formation. (**A**) Temporal profile of relative miR-218 expression levels in Bm7brmx2 cells overexpressing tet-on pri-mir-218 after doxycycline (2 μg/mL) treatment. (**B**) Western blot analysis of CDCP1 in Bm7brmx2 cells overexpressing tet-on pri-mir-218 four days after doxycycline treatment. EF1α was used as an internal control. (**C**) Migration ability of Bm7brmx2 cells overexpressing tet-on pri-mir-218 on the fourth day after doxycycline treatment using time-lapse video microscopy. (**D**) Relative survival of Bm7brmx2 cells overexpressing tet-on pri-mir-218 in anchorage-free culture conditions after doxycycline induction. (**E**) Quantitation of tumor spheres on days 6 and 12 after doxycycline induction. (**F**) Size of tumor spheres on day 9 after doxycycline induction. *P < 0.05. **P < 0.01.

To examine whether restoration of miR-218 would inhibit the tumorigenesis of cancer cells, we constructed an inducible tet-on plasmid to express pri-mir-218. As shown in Fig. 5A, the expression levels of miR-218 were induced in the presence of doxycycline. Similarly, the amount of CDCP1 protein was decreased on day 4 post-induction with doxycycline (Fig. 5B). Cell mobility was significantly (*P* < 0.01) decreased in Bm7 cells under miR-218 induction in a time-lapse migration assay (Fig. 5C). To further evaluate the function of CDCP1 in anoikis resistance^10^, the survival of Bm7 cells with miR-218 induction was significantly reduced in anchorage-free culture conditions (Fig. 5D). In addition, the tumor-initiating cell (TIC) formation (Fig. 5E) and sphere size (Fig. 5F) were significantly reduced in miR-218 induced cells. Thus, overexpression of miR-218 did inhibit oncogenetic ability in lung cancer cells, such as migration, anoikis resistance, and tumor sphere formation.

### miR-218 reduces lung cancer metastasis and improves animal survival

**Figure 6:**
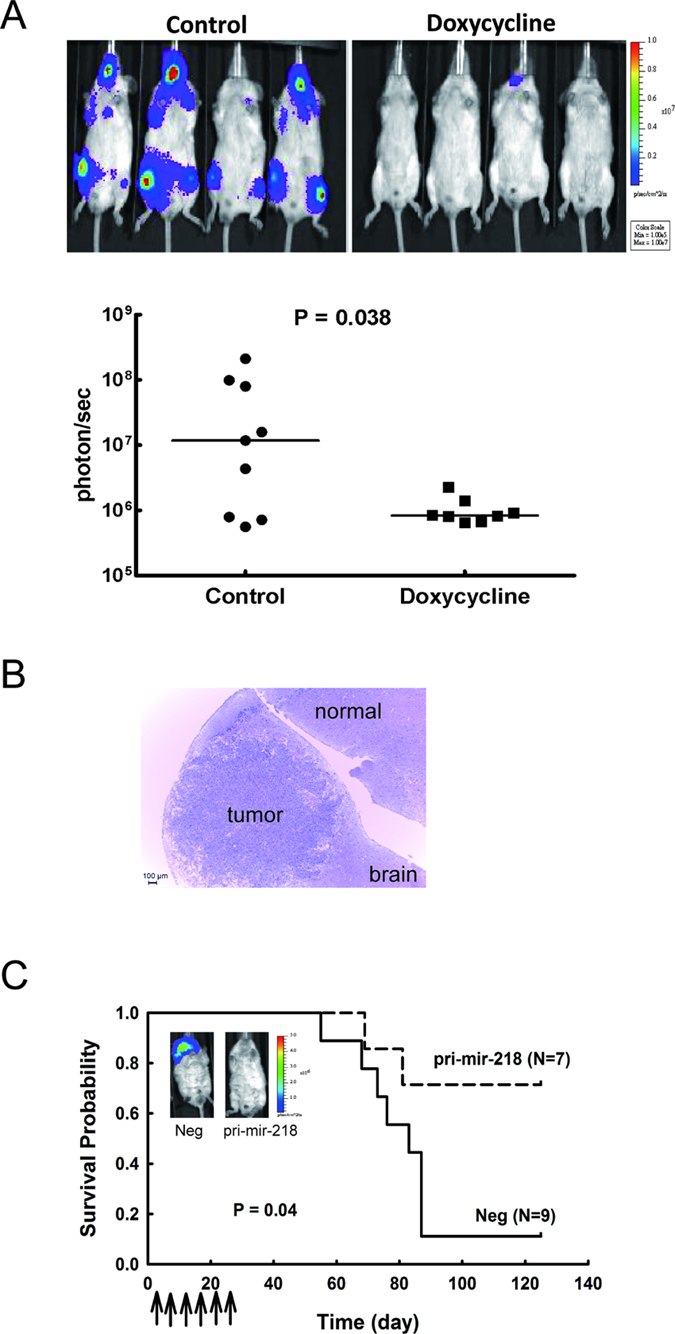
Overexpression of miR-218 inhibits tumor metastasis and increases survival in mice bearing brain-metastatic lung cancer cells. (**A**) Induction of miR-218 inhibited tumor metastasis in mice bearing brain-metastatic lung cancer cells. Bm7brmx2 cells with lentiviral tetracycline-inducible pri-mir-218 plasmid were injected intracardially into SCID mice. The mice were treated with doxycycline (2 mg) to induce miR-218 expression by intraperitoneal injection once a day for 9 days. Quantitative signals from tumors in mice on day 28 are shown below the intensity images and analyzed by Mann-Whitney test. Each spot represents the signal from one mouse. (**B**) Representative hematoxylin and eosin staining of brain tissue from control group. The metastatic lung tumor cells were identified in the brain of control group. Scale bar: 100 μm. (**C**) Kaplan-Meier survival analysis of SCID mice expressing miR-218. SCID mice received intracardial injection of 5 × 10^4^ Bm7brmx2 cells. Starting one day after cancer cell injection, tumor bearing mice were treated with 25 μg of pri-mir-218-liposome complexes by i.v. injection twice a week for 3 weeks. Arrows indicate the time points of pri-mir-218-liposome therapy. N = 9 in the negative control (Neg) group and N = 7 in the pri-mir-218 group. The insert of bioluminescent image was detected on day 83.

To further evaluate the antitumor effects of miR-218 *in vivo*, we established a metastatic lung tumor animal model by intracardially inoculating human lung cancer cells, Bm7brmx2 with inducible expression of pri-mir-218, into severe combined immunodeficiency (SCID) mice. Luciferase activity was measured to reflect the tumor size with an IVIS imaging system. High tumor metastasis was observed in the control group as compared to the doxycycline group on day 28 after tumor injection (Fig. 6A,B). Also, we used another xenograft animal model to determine the therapeutic effect of miR-218 plasmids. One day after tumor inoculation, tumor-bearing mice were treated with systemic delivery of liposomal DNA complex containing pri-mir-218 twice a week for 3 weeks. The survival curve demonstrated that pri-mir-218 plasmids significantly prolonged the survival time compared with the control group (Fig. 6C). Taken together, our results demonstrate that miR-218 provides an antitumor effect in inhibiting brain metastases of lung tumors in mice.

## Discussion

Here we demonstrated that ADAM9 could inhibit the expression of miR-218 and that miR-218 can directly bind to the 3′-UTR of *CDCP1*. Down-regulation of miR-218 led to CDCP1 overexpression, which promoted malignancy of lung cancer cells. Restoring the miR-218 levels in lung cancer cells provided an antitumor effect, as shown by reduction of anoikis resistance, suppression of tumor sphere formation, and decreased metastasis. Although several reports have documented that up-regulation of CDCP1 expression was associated with disease progression in different human tumor types^20^, our data are the first study to report that *CDCP1* is regulated by miR-218, and hence provide a novel regulatory mechanism of miR-218 involved in the ADAM9-CDCP1 axis in lung adenocarcinoma.

Although there are three potential targeting sites in the 3′-UTR of *CDCP1*, in this study, we demonstrated that miR-218 can directly bind to two of them (1,840–1,860 bp and 2,951–2,971 bp, labeled as WTA and WTC in Fig. 3) using luciferase reporter assays. Interestingly, the WTA binding site (1,840–1,860 bp) exists only in primates and the WTC binding site (2,951–2,971 bp) contains evolutionarily conserved binding sites for miR-218 in mice. Furthermore, the WTA binding site seems to play a more important role than the WTC site in miR-218 regulation, because the suppressive effect of miR-218 in the WTA mutant is strongly reversed and consistently observed in three lung cancer cell lines (Fig. 3).

Since miRNA can down-regulate multiple targets by interacting with different mRNAs^21^, miRNA is an attractive approach for cancer treatment, if the key miRNAs regulating multiple important oncogenes are identified. Hypothetically, since oncogenes are over-expressed only in tumor cells, administration of miRNA would discriminately block the mRNA of oncogenes in tumor cells without affecting normal cells. Recently, miR-218 attracts scientists’ attention due to significant repression in several kinds of cancer tissues and correlation with poor prognosis^17^. Notably, the ectopic expression of miR-218 can act as a tumor suppressor by targeting genes related to proliferation, apoptosis, and invasion^17^. For example, miR-218 can suppress nasopharyngeal cancer progression through down-regulation of *BIRC5* (survivin) and the SLIT2 (slit guidance ligand 2)-ROBO1 (roundabout guidance receptor 1) pathway^22^. miR-218 may also target mTOR component *RICTOR* (Rictor) in oral cancer^23^. In addition, it can down-regulate *PXN* (paxillin) to suppress relapse in non-small cell lung cancer^24^ and down-regulate *CDH2* (N-cadherin) expression^16^, which is associated with non-small cell lung cancer (NSCLC) metastatic spread to the brain^25^. In addition, emerging evidence has implicated that tumor-initiating cells (TIC) are associated with the conversion of early stage tumors into invasive malignancies accompanied by increased cancer cell motility and invasion^26^. For instance, miR-218 is under-expressed in CD44^+^ prostate cancer cells^27^, which are enriched in TICs^28^. Also, it has been reported that miR-218 reduced self-renewal capacity in glioma stem-like cells by targeting stem cell promoting oncogene *BMI1*(BMI1 proto-oncogene, polycomb ring finger)^29^. Consistent with these findings, we demonstrated that miR-218 influences TIC formation in lung cancer. These studies support our findings that miR-218 indeed suppresses many oncogenic genes and has great anti-tumorigenic potential. Thus, miR-218 could be used as a therapeutic agent for lung cancer. Restoration of miR-218 expression might be a new therapeutic strategy to prevent lung cancer cells metastasizing to brain.

## Methods

### Cell culture and reagents

Human lung cancer cell lines (CL1-0, F4, Bm7, Bm7brmx2, A549, and H1299) were used in this study as previously described^16^. Cell lines were cultured within 6 months from the established cell bank to keep their ability to form lung cancer tumors in SCID mice. All cell lines were clean from *Mycoplasma* contamination. The following antibodies were used in western blots: anti-CDCP1 (ab1377, Abcam, Cambridge, MA, USA), anti-elongation factor 1 α (EF1α, #05-235, Millipore, Billerica, MA, USA), anti-ADAM9 (MAB939, R&D Systems, Minneapolis, MN, USA), and anti-ADAM9 (#2099, Cell Signaling, Danvers, MA, USA).

### Quantitative reverse transcription PCR in clinical specimens

Quantitation of miR-218 was performed as previously described^16^. The following primers were used for mRNA detection: *CDCP1*: 5′-ttaccccaaggactgtggac-3′ (forward), 5′-cgagggcagacagcagtaa-3′ (reverse). *ADAM9*: 5′-cccccaaattgtgagactaaag-3′ (forward), 5′-tccgtccctcaatgcagtat-3′ (reverse). The relative expression level of *CDCP1* and *ADAM9* mRNA was normalized against *GAPDH* (glyceraldehyde 3-phosphate dehydrogenase) mRNA. The lung tumor specimens were obtained from patients admitted to China Medical University Hospital (CMUH). Written informed consent was obtained in compliance with protocols and the experiments were carried out in accordance with the approved guidelines by the CMUH Institutional Review Board.

### Plasmids, transfection, and generation of stable cell lines

The plasmid containing sequences of primary miR-218 was constructed as previously described^16^. The *CDCP1* 3′-UTR was amplified from leukocyte genomic DNA and inserted into the pMIR-REPORT miRNA Expression Reporter Vector (Applied Biosystems, Carlsbad, CA, USA) by adding the *Spe*I and *Mlu*I sites. Three miR-218 binding sites were predicted in the *CDCP1* 3′-UTR using miRSystem^19^, located at 1,840–1,860 bp, 2,100–2,119 bp, and 2,951–2,971 bp relative to the transcription start site. Mutations of the miR-218 binding sites in the *CDCP1* 3′-UTR or mutations of the primary miR-218 sequence were made using the QuikChange Site-Directed Mutagenesis Kit (Agilent Technologies, Santa Clara, CA, USA) according to the manufacturer’s protocol. These reporter plasmids containing the *CDCP1* 3′-UTR, primary miRNA, and *Renilla* luciferase sequences were co-transfected into the indicated cells as previously described. Luciferase activity was measured using the Dual-Luciferase Reporter Assay System (Promega, Madison, WI, USA). Lung cancer cells expressing doxycycline-inducible pri-mir-218 were generated by infecting Bm7brmx2 cells with a lentiviral tet-on-pri-mir-218 plasmid to generate stable cell lines, as previously described^16^.

### Time-lapse migration assay

This assay was conducted as previously described^15^. Briefly, cells were cultured on collagen-coated dishes (10 μg/mL, 3 mL) in serum-free media. The migration of cells was captured with CCD video cameras (AxioCam MRm, Zeiss) at 20 min intervals for a total 16 hours by inverted microscopes (Axio Observer Z1, Zeiss). Accumulated migration distance was determined by using the Track Point function of Image J.

### Sphere formation assay

Lung cancer cells (1,000 cells per mL) were plated on 1% agarose coated dishes and cultured in DMEM/F12 media supplemented with N-2 (100×, Life Technologies), 10 ng/mL human recombinant bFGF, 10 ng/mL EGF, and 1% antibiotic (Penicillin-Streptomycin, Life Technologies). The number of spheres was counted on different days under doxycycline treatment (2 μg/mL, Sigma, Shanghai, PRC).

### Lung cancer animal model

All animal experiments were carried out under protocols approved by the Institutional Animal Care and Use Committee of China Medical University and Hospital. Lung cancer cells with stable luciferase expression (5 × 10^4^ cells) were injected intracardially into 6–8 week-old SCID mice (BioLASCO, Taiwan) and imaged by an IVIS Spectrum Imaging system (Xenogen, Hopkinton, MA, USA) under specific pathogen-free conditions. The therapeutic protocol was similar to that of a previous study^30^. Briefly, the mice received intravenous injections of 100 μL of DNA:liposome (HLDC) complex containing 25 μg of plasmids each time, twice a week and for a total of 6–8 times.

### Statistical analysis

Comparisons between the analyzed parameters were performed using Student’s t-test or two-way AVOVA for continuous variables. All statistical tests were two-sided. Survival curves were obtained by the Kaplan–Meier method. Statistical significance was set for all tests at *P* < 0.05.

## Additional Information

**How to cite this article**: Chiu, K.-L. *et al.* ADAM9 enhances CDCP1 protein expression by suppressing miR-218 for lung tumor metastasis. *Sci. Rep.* **5**, 16426; doi: 10.1038/srep16426 (2015).
